# Dysregulation of the Extracellular Matrix in the Synaptic Pathology of Parkinson’s Disease: Molecular Mechanisms and Novel Therapeutic Approaches

**DOI:** 10.3390/life16061007

**Published:** 2026-06-15

**Authors:** Carmen Rubio, Ricardo Pérez-Rubio, Javier Pérez-Villavicencio, Norma Serrano-García, Ángel Lee, Leticia Granados-Rojas, Martha Tena-Suck, Moisés Rubio-Osornio

**Affiliations:** 1 Neurophysiology Department, National Institute of Neurology and Neurosurgery, Mexico City 14269, Mexico; mrubio@innn.edu.mx (C.R.); r.prf@lasallistas.org.mx (R.P.-R.); javierperezvillavicencio@gmail.com (J.P.-V.); norma.serrano@innn.edu.mx (N.S.-G.); martha.tena@innn.edu.mx (M.T.-S.); 2 Mexican Faculty of Medicine, La Salle University, Mexico City 14000, Mexico; 3 Department of Electrical Engineering, Basic Sciences and Engineering Division, Metropolitan Autonomous University, Iztapalapa Campus, Mexico City 09340, Mexico; 4 Public Health Intelligence Unit, National Institute of Public Health, Cuernavaca 62100, Mexico; dr_angel_lee@yahoo.de; 5 Laboratory of Biomolecules and Infant Health, National Institute of Pediatrics, Mexico City 04530, Mexico; granados_2000@yahoo.com.mx; 6 Pathology Department, National Institute of Neurology and Neurosurgery, Mexico City 14269, Mexico; 7 Neurochemistry Department, National Institute of Neurology and Neurosurgery, Av. Insurgentes Sur 3877, Mexico City 14269, Mexico

**Keywords:** Parkinson’s disease, extracellular matrix, α -synuclein, matrix metalloproteinases, perineuronal nets, synaptic dysfunction, neuroinflammation, biomarkers

## Abstract

Parkinson’s disease (PD) is defined by the progressive degeneration of dopaminergic neurons within the substantia nigra and the pathological accumulation of α-synuclein (α-syn) aggregates. Beyond these intracellular hallmarks, the extracellular matrix (ECM) has emerged as an active regulator of synaptic dysfunction, neuroinflammation, and disease progression. Recent multi-omics evidence, including transcriptomic and proteomic profiling of post-mortem tissue and iPSC-derived neurons, demonstrates consistent dysregulation of ECM components across both sporadic and genetic PD subtypes—typified by downregulation of basement membrane collagens (COL4A) and integrin signaling (ITGB1), alongside upregulation of matrix metalloproteinases (MMPs). These alterations destabilize perineuronal nets (PNNs), allow prion-like α-syn propagation, and promote glial activation through TLR-mediated signaling. Circulating ECM-derived neoepitopes (C1M, C4M) may complement neurofilament light chain as prognostic biomarkers of disease progression, although prospective validation remains necessary. Pharmacological strategies targeting MMP activity (e.g., doxycycline), integrin–FAK signaling (ATN-161), and MMP–TIMP balance (mesenchymal stem cells) represent emerging therapeutic avenues, though clinical evidence in PD remains limited. This review synthesizes current evidence on ECM dysregulation in PD and discusses its implications for biomarker development and disease-modifying intervention.

## 1. Introduction

Parkinson’s disease (PD) is the second most prevalent neurodegenerative disorder worldwide, affecting over 10 million individuals, a figure expected to rise with global population aging [1,2,3]. Clinically, PD is defined by the progressive degeneration of dopaminergic neurons in the *Substantia Nigra pars compacta* (*SNpc*) and dysfunction of the nigrostriatal pathway, resulting in cardinal motor manifestations such as bradykinesia, rigidity, and resting tremor, alongside diverse non-motor symptoms [4,5,6,7]. The pathological hallmark of PD is the abnormal accumulation of alpha-synuclein (α-syn) within Lewy bodies and Lewy neurites [8]. Chronic neuroinflammatory cascades mediated by microglia and astrocytes also contribute to early synaptic dysfunction and disease progression [9]. Research on PD pathogenesis has traditionally focused on intracellular mechanisms, including mitochondrial dysfunction, oxidative stress, and impaired protein degradation through the ubiquitin–proteasome system and autophagy [10]. Although these studies have clarified key aspects of PD biology, they do not fully explain the clinical heterogeneity or the early synaptic alterations that precede neuronal loss [11,12,13]. More recent work suggests that trans-neuronal propagation of misfolded α-syn aggregates is central to disease progression [14,15,16]. Within this framework, the extracellular matrix (ECM) of the central nervous system (CNS) has gained attention as a dynamic regulator of neuronal connectivity, synaptic stability, and neuroinflammatory signaling [17,18].

The neural ECM is a structurally organized and functionally diverse network composed of collagens, laminins, glycoproteins, and chondroitin sulfate proteoglycans, which regulate cell adhesion, integrin signaling, and the spatial compartmentalization of synaptic microdomains [19,20,21]. Under physiological conditions, these constituents support the assembly of perineuronal nets (PNNs) that stabilize mature neural circuits and modulate activity-dependent synaptic plasticity [22]. ECM structural integrity also acts as a barrier against the intercellular dissemination of aggregated proteins such as α-syn [23]. This barrier function relies on at least three complementary mechanisms: (i) the dense meshwork of chondroitin sulfate proteoglycans and hyaluronan sterically hinders the diffusion of large oligomeric species [22,24]; (ii) perineuronal nets enwrap specific neuronal populations and limit the lateral mobility of membrane-associated aggregates [22]; and (iii) ECM components such as laminin and collagen IV bind directly to cell-surface receptors (e.g., integrins, dystroglycan), sequestering extracellular α-syn and reducing its availability for uptake by neighboring cells [19,20]. Consequently, degradation of these structures by MMPs removes physical restraint on prion-like propagation.

Recent multi-omics studies integrating transcriptomic and proteomic analyses of post-mortem brain tissue, experimental models, and patient-derived induced pluripotent stem cell (iPSC) neurons have shown widespread dysregulation of ECM components in both genetic and sporadic forms of PD [24,25,26,27,28,29,30]. A recent systematic review of ECM-related transcriptional and proteomic alterations in PD [30] provides a synthesis of these observations, which the present narrative review extends by integrating mechanistic, biomarker, and therapeutic dimensions. These studies frequently report downregulation of structural collagens and integrin subunits together with upregulation of matrix metalloproteinases [31]. ECM disruption alters synaptic signaling and enables prion-like α-syn propagation [32], and ECM degradation products detected in the cerebrospinal fluid of patients with PD correlate with markers of synaptic dysfunction and clinical progression [33]. Together, this evidence challenges the view of the ECM as a passive scaffold and supports its active role in modulating synaptic vulnerability and PD pathogenesis [34]. Whereas previous systematic reviews have catalogued ECM-related transcriptional and proteomic alterations in PD [30,35], the present narrative review extends this body of work along three complementary axes that remain underdeveloped in the current literature. First, it integrates ECM dysregulation with synaptic-level mechanisms, specifically integrin β1–FAK/Src signaling, GluA1-AMPA receptor anchoring, and perineuronal net stability around parvalbumin-positive interneurons. Second, it critically appraises ECM-derived neoepitopes (C1M, C4M) as candidate prodromal and prognostic biomarkers, including their comparative performance against neurofilament light chain and their limitations regarding CNS specificity. Third, it provides a stratified evaluation of ECM-targeted therapeutic strategies in PD organized by level of clinical evidence, distinguishing experimental concepts from preclinical findings and from interventions with early human data. This integrative framing positions the ECM not merely as a dysregulated compartment but as a mechanistic node connecting synaptic vulnerability, biomarker development, and disease-modifying therapy. Establishing whether ECM dysregulation is a cause or a consequence of neurodegeneration is challenging in cross-sectional human studies. Nevertheless, several lines of evidence argue for an early pathogenic role: (i) iPSC-derived dopaminergic neurons carrying PD-linked mutations exhibit ECM transcriptome changes long before overt cell death [36,37]; (ii) in the A53T mouse, PNN fragmentation precedes significant dopaminergic loss [38,39]; and (iii) circulating ECM neoepitopes are elevated in prodromal cohorts, correlating with subsequent motor progression independently of baseline neuronal injury markers [40,41]. These observations suggest that ECM remodeling can initiate synaptic instability and glial activation, which in turn accelerate α-syn pathology and neurodegeneration, forming a feed-forward loop. Future longitudinal studies with inducible genetic models will be needed to definitively resolve causality.

## 2. Transcriptomic and Proteomic Foundations of Extracellular Matrix Dysregulation in PD

High-throughput transcriptomic profiling (RNA-seq) of *SNpc* tissue from PD cohorts, including specimens from the Harvard Brain Tissue Resource Center and the NINDS NeuroBioBank, has revealed widespread transcriptomic changes in PD, with many differentially expressed genes mapping to ECM pathways [26,40]. Structural basement membrane collagens (COL4A1, COL4A2, COL6A1/2) are consistently downregulated, while matrix metalloproteinases (MMP9, MMP13, MMP14) and their endogenous inhibitors TIMP1 and TIMP3 show altered expression, indicating a shift toward ECM degradation in dopaminergic regions [31,42]. Gene set enrichment analyses (GSEA) consistently identify &quot; ECM structural constituent &quot; and &quot; ECM organization &quot; among the most downregulated biological processes, supporting progressive disassembly of extracellular scaffolding in vulnerable nigrostriatal circuits [26,40,43,44]. Single-cell RNA sequencing (scRNA-seq) of dopaminergic neurons derived from iPSCs, including isogenic disease-control platforms [45], confirms these findings and shows reductions in transcripts linked to focal adhesion and synaptic anchoring, particularly ITGB1 and VCL [36,37]. At the protein level, integrin β1 clustering activates focal adhesion kinase (FAK) through autophosphorylation at Tyr397, which recruits Src family kinases and triggers a cascade that phosphorylates key postsynaptic scaffolds such as p130Cas and paxillin. This signaling stabilizes the actin cytoskeleton and promotes the synaptic retention of AMPA receptors by facilitating CaMKII-dependent phosphorylation of GluA1 at Ser831. Loss of ITGB1 therefore weakens this entire structural-signaling module, leading to dendritic simplification, reduced density of PSD95-positive excitatory synapses, and impaired excitatory transmission [46,47]. Proteomic studies using LC-MS/MS of cerebrospinal fluid (CSF) from patients with early-stage PD show persistent activation of ECM remodeling pathways [48], with elevated concentrations of collagenolytic neoepitopes (C1M and C4M) consistent with increased metalloproteinase activity in the CNS [49]. These ECM-derived fragments correlate with phosphorylated α-syn (Ser129) and indices of synaptic injury, suggesting their potential use as prodromal biomarkers of disease progression [50,51]. Together, these results reinforce the view that ECM breakdown is an early, mechanistically relevant event in PD pathogenesis that may precede overt synapse loss and neurodegeneration as summarized in Table 1 [17,52].

## 3. Evidence from Animal Models and Human Studies

Experimental evidence from animal models and human neuropathological studies supports ECM dysregulation as a central contributor to the pathophysiology of PD, closely linked to synaptic dysfunction, neuroinflammatory responses, and progressive neurodegeneration [62,75]. In transgenic mouse models overexpressing mutant α-syn, particularly the A53T variant, early disruption of PNNs has been documented. Histological analyses using Wisteria floribunda agglutinin staining show fragmentation of PNNs in the striatum and cortex, with a 50–60% reduction in ECM-associated cell labeling by six months of age [38,39]. These structural changes coincide with progressive synaptic degeneration, reduced dopaminergic projections from the substantia nigra, and increased propagation of pathogenic α-syn aggregates [76,77]. Functional studies show that enzymatic ECM remodeling increases neuronal vulnerability: genetic ablation or pharmacological inhibition of matrix metalloproteinase-9 partially restores dopaminergic neuron integrity, increasing tyrosine hydroxylase–positive cell counts in the *SNpc* and reducing synaptic degeneration in α-syn transgenic models [64,65]. Together, these findings implicate metalloproteinase-mediated ECM breakdown as a mechanism of dopaminergic neurodegeneration [66]. Toxin-based models, particularly the MPTP mouse model, reproduce key features of sporadic PD. MPTP exposure induces ECM remodeling within the nigrostriatal axis, with accumulation of fragmented hyaluronic acid and other ECM degradation products in the striatal extracellular space [55,56]. Studies in both mouse and non-human primate MPTP models show that MMP-9 contributes to inflammatory glial activation and nigrostriatal degeneration [63]. These ECM fragments trigger innate immune signaling via pattern-recognition receptors, particularly Toll-like receptors, on astrocytes and microglia, promoting the neurotoxic A1 astrocyte phenotype with sustained pro-inflammatory mediator release and impaired synaptic maintenance [57,78,79]. These observations anticipate a self-reinforcing cycle linking ECM breakdown, glial activation, and α-syn accumulation that is developed mechanistically in Section 6.

Neuropathological analyses of human post-mortem tissue show extensive ECM remodeling in advanced PD, particularly within motor and cognitive circuits [26,40]. Aggrecan and other key PNN constituents are reduced in the hippocampus, prefrontal cortex, and other limbic and cortical regions [22,54]. Because aggrecan is a principal proteoglycan supporting synaptic stability and network integrity [17], its depletion contributes to synaptic destabilization and impaired plasticity in memory and learning domains. Clinically, these ECM alterations are associated with cognitive decline and reduced neuropsychological performance, particularly in PD dementia [12]. Multi-omics studies in human cohorts further support the link between ECM dysregulation and PD pathogenesis. Transcriptomic and genetic datasets from consortia such as the Parkinson Progression Marker Initiative (PPMI) [80] and the UK Biobank consistently show alterations in ECM organization, focal adhesion, and ECM receptor interaction pathways in both sporadic and familial PD [26,35]. Single-nucleus RNA sequencing also identifies microglial subpopulations with an “ECM-high” transcriptional signature, characterized by elevated metalloproteinases, integrins, and pro-inflammatory mediators, suggesting neuroimmune-ECM crosstalk in disease progression [40,81]. Overall, evidence from animal models and human neuropathology positions ECM instability as a central determinant of synaptic vulnerability, prion-like α-syn spread, and neuroinflammation in PD [40,64,78].

## 4. Emerging Biomarkers Originating from the Extracellular Matrix

Sensitive biomarkers that can track PD progression during prodromal stages, when synaptic dysfunction precedes overt neurodegeneration, remain an unmet need [26,40]. ECM-derived neoepitopes, generated by proteolytic cleavage of matrix constituents through matrix metalloproteinase activity, have emerged as candidate biomarkers of active remodeling and neurodegenerative processes [41,64]. Quantification of circulating collagen fragments such as C1M and C4M in serum using enzyme-linked immunosorbent assays enables detection of altered ECM turnover in neurological disorders [49,82]. In PD, these biomarkers track disease trajectory longitudinally, reflecting synaptic remodeling and basement membrane integrity [41]. Longitudinal cohort studies support the clinical utility of ECM-derived biomarkers: elevated baseline C1M and C4M levels in cohorts such as ICICLE-PD independently predict accelerated clinical deterioration, as assessed by the Unified Parkinson’s Disease Rating Scale [83,84]. Increased serum concentrations of these neoepitopes correlate with greater annual increases in UPDRS scores, supporting their value as early indicators of rapid disease progression [26,66]. Comparative analyses show that ECM fragments provide complementary or, in some studies, stronger prognostic information than established neurodegeneration biomarkers such as neurofilament light chain [79,82,85], with receiver operating characteristic analyses showing improved predictive performance for ECM-related measures [79,82]. Importantly, ECM remodeling biomarkers capture upstream pathophysiological processes that precede irreversible neuronal injury. The identification of patient subgroups with persistently elevated ECM biomarkers, termed “ECM-high progressors”, has advanced prognostic stratification by identifying individuals with aggressive disease trajectories and sustained extracellular remodeling activity [86,87]. Elevated circulating C1M is associated with advanced neuropathological stages and a greater burden of non-motor symptoms, including cognitive impairment and autonomic dysfunction [40,41]. These findings support ECM-based biomarkers as useful tools for risk stratification and for identifying patients with accelerated progression. From a translational perspective, ECM-derived biomarkers reflect active, upstream remodeling processes rather than end-stage neuronal damage [66]. ECM-based stratification strategies may therefore help optimize patient selection and enrichment in clinical trials targeting neuroinflammation, synaptic resilience, or ECM modulation [26,41].

## 5. Synaptic Remodeling and Pathological Dissemination of α-Syn

**Figure 1:**
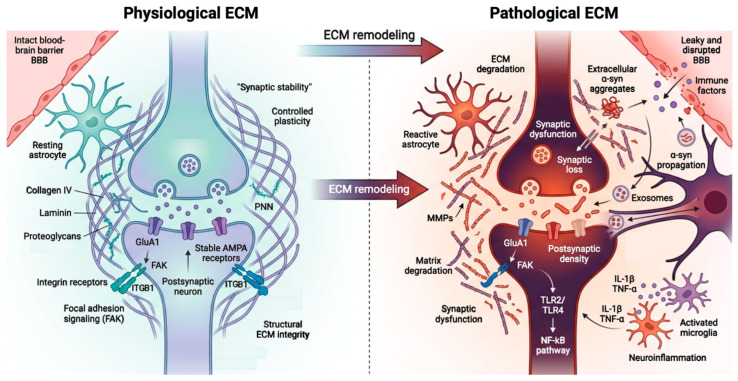
**Structural and functional remodeling of the extracellular matrix in the synaptic microenvironment of Parkinson’s disease.** Schematic comparison of the synaptic microenvironment under physiological and pathological conditions. (**Left**): Structural ECM components—collagen IV, laminin, and proteoglycans—together with integrin β1 (ITGB1)–focal adhesion kinase (FAK) signaling, sustain stable GluA1-AMPA receptor anchoring, controlled synaptic plasticity, and an intact blood–brain barrier (BBB). (**Right**): In PD, MMP–driven ECM degradation disrupts ITGB1–FAK signaling, destabilizes the postsynaptic density, and compromises BBB integrity. ECM remodeling promotes the prion-like propagation of misfolded α-synuclein (α-syn) through extracellular release and exosome-mediated transfer. ECM-derived signals engage Toll-like receptors 2 and 4 (TLR2/TLR4) on glial cells, triggering NF-κB activation, reactive astrogliosis, microglial activation, and pro-inflammatory cytokine release (IL-1β, TNF-α). Created in BioRender. Perez Rubio R. (2026) https://BioRender.com/m0c60pz (accesed on 26 May 2026).

The ECM is a key regulator of synaptic plasticity and neuronal circuit stability, acting through integrin-β1 and focal adhesion complexes that include focal adhesion kinase (FAK) and Src family kinases [17,20]. Under physiological conditions, this signaling axis supports long-term potentiation via calcium-dependent kinases such as CaMKII and promotes phosphorylation of postsynaptic receptor subunits, maintaining excitatory synaptic transmission [88,89]. The structural and functional contrasts between the physiological and pathological ECM in the synaptic microenvironment of PD are summarized in Figure 1.

Activation of these pathways recruits and stabilizes AMPA receptor subunits, particularly GluA1, within the postsynaptic density, supporting synaptic strength and plasticity [90,91]. In addition to local synaptic effects, integrin-FAK signaling engages downstream survival and plasticity pathways. FAK can activate the Ras-ERK cascade, promoting CREB phosphorylation and the transcription of synaptic plasticity-related genes such as BDNF [92]. Likewise, FAK-dependent PI3K-Akt signaling supports neuronal survival and the maintenance of synaptic spine density [93]. Dysregulation of these pathways in PD further compromises the capacity of neurons to sustain long-term potentiation and to counteract excitotoxic stress, thereby exacerbating synapse loss. In PD, disruption of integrin–FAK/Src signaling destabilizes postsynaptic architecture and reduces anchoring of GluA1-containing receptors, impairing excitatory neurotransmission and synaptic resilience [17,40]. Structural alterations of the ECM further promote the pathological spread of α-syn through mechanisms resembling prion-like propagation across interconnected neuronal networks [85]. ECM fragmentation and instability of perineuronal nets alter the extracellular space, modulating extracellular vesicle trafficking and intercellular communication [22,64]. These changes facilitate the release and uptake of pathogenic α-syn species via exosomes enriched in ECM remodeling proteins [94,95]. Altered ECM dynamics also promote the formation of tunneling nanotubes, enabling direct intercellular transfer of α-syn aggregates and their synapse-to-synapse spread along neuronal circuits [96,97]. Human astrocytes can also transfer aggregated α-syn to neighboring astrocytes via direct contact and tunneling nanotubes when their lysosomal degradation capacity is exceeded [98]. During neuroinflammatory states, matrix metalloproteinases degrade perineuronal net structures that surround parvalbumin-positive interneurons [22,66]. This enzymatic cleavage exposes inhibitory interneurons to oxidative stress and extracellular toxins, impairing local modulation of inhibitory tone within neural circuits [22,64]. Loss of this structural protection promotes excitotoxic conditions that destabilize synaptic networks and accelerate dopaminergic degeneration within the nigrostriatal pathway [40,81]. Reduction in basement membrane collagens, particularly type IV collagen, further weakens synaptic microenvironment stability by disrupting basement membrane integrity and altering adhesion complexes that support presynaptic vesicle docking and neurotransmitter release [42,64]. ECM instability may therefore facilitate altered vesicular trafficking and the pathological release of toxic α-syn species from presynaptic terminals into the extracellular space [40,85]. Overall, these mechanisms link synaptic dysfunction with proteopathic spread, making neuronal circuits progressively more vulnerable as PD advances [42,64].

## 6. Neuroinflammatory Responses Driven by ECM Remodeling

Pathological remodeling of the ECM strongly influences neuroinflammatory cascades in PD, linking synaptic injury, glial activation, and α-syn propagation [64,81]. Degradation of structural ECM components releases soluble fragments that act as damage-associated molecular patterns (DAMPs), initiating innate immune signaling within the CNS [99]. Among these ECM-derived DAMPs, biglycan and low-molecular-weight hyaluronic acid engage pattern recognition receptors such as Toll-like receptor 2 (TLR2) and Toll-like receptor 4 (TLR4) on microglia [58,59]. Engagement of these receptors activates intracellular signaling pathways, including the NF-κB and MAPK cascades, leading to upregulation of reactive oxygen species and pro-inflammatory cytokines such as IL-1β, TNF-α, and IL-6 [60,61]. This neuroinflammatory environment increases oxidative stress and impairs synaptic function within the nigrostriatal pathway. ECM-driven inflammatory signals can directly modulate α-syn pathology: exposure of dopaminergic neurons to inflammatory cytokines and oxidative stress promotes phosphorylation of α-syn at Ser129, a post-translational modification associated with pathological aggregation and Lewy body formation [100,101]. The extracellular release of α-syn aggregates activates microglia, amplifying inflammatory signaling and further ECM degradation [102,103]. Matrix remodeling, glial activation, and α-syn propagation thus reinforce one another in a positive feedback loop: ECM-derived DAMPs engage TLR2/TLR4 to drive glial activation; activated glia release MMPs that further degrade ECM and disrupt synaptic anchoring; and the resulting extracellular environment facilitates α-syn release, uptake, and aggregation, which in turn perpetuates microglial activation. This self-reinforcing cycle represents a unifying mechanistic framework for the convergence of ECM dysregulation, neuroinflammation, and proteopathic spread in PD.

Genetic determinants can intensify this neuroinflammatory ECM-glial axis by altering vesicular trafficking and immune signaling. For example, pathogenic LRRK2 variants, particularly the G2019S mutation, increase phosphorylation of Rab GTPases involved in vesicle transport, altering astrocyte and microglial secretion of inflammatory mediators and ECM components [104,105,106]. This produces elevated levels of inflammatory ECM fragments in the extracellular space, shifting microglial polarization toward neurotoxic phenotypes and worsening reactive astrogliosis [78]. Experimental models using patient-derived iPSC brain organoids support these mechanisms: pharmacological blockade of TLR signaling reduces glial activation and cytokine production, supporting an active contribution of ECM-derived inflammatory cues to neurodegenerative cascades [26,81]. Together, these findings implicate ECM remodeling as a central driver of neuroinflammation in PD, linking extracellular structural damage, innate immune activation, and progressive neuronal dysfunction [64,66].

## 7. Convergence with Traditional Pathways in Parkinson’s Disease

ECM disruption intersects with canonical pathogenic processes in PD, including mitochondrial dysfunction, impaired proteostasis, and chronic neuroinflammation [4,107]. Structural ECM elements influence the spatial organization of intracellular signaling networks, including pathways relevant to mitochondrial quality control through integrin-mediated mechanotransduction and cytoskeletal coupling [20]. A proposed mechanistic model linking collagen IV to PINK1/Parkin-mediated mitophagy posits that type IV collagen, through its binding to integrin α1β1 and dystroglycan complexes, couples the extracellular matrix to the actin cytoskeleton and, via cytoskeletal continuity, to the peri mitochondrial scaffold. Within this model, ECM–cytoskeletal coupling would help stabilize PINK1 accumulation on the outer membrane of depolarized mitochondria and facilitate Parkin recruitment and subsequent ubiquitination of outer-membrane substrates [53,108]. Loss of basement membrane collagens, particularly type IV collagen, would therefore be expected to disrupt this scaffold, impair mitophagic flux, and contribute to the accumulation of dysfunctional organelles in dopaminergic neurons. It should be emphasized, however, that this collagen IV integrin cytoskeleton PINK1/Parkin axis remains a working model: the individual molecular interactions are supported by evidence in non-neuronal systems, but the integrated cascade has not been formally demonstrated in dopaminergic neurons of PD models, and direct experimental validation in the parkinsonian context is still required.

**Figure 2:**
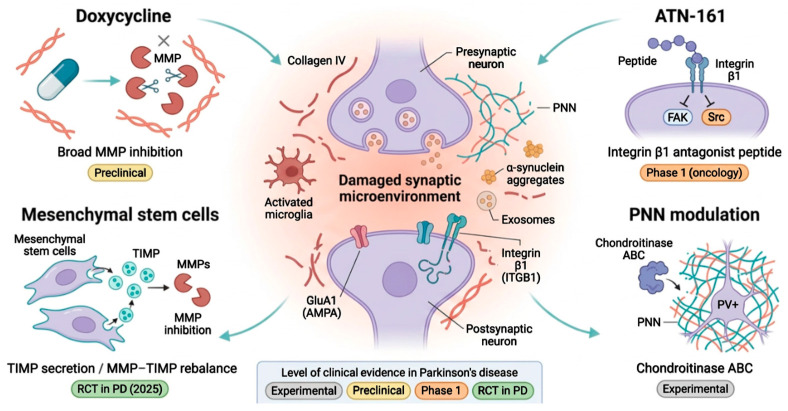
**ECM-targeted therapeutic strategies in Parkinson’s disease.** Schematic overview of four therapeutic approaches converging on the damaged synaptic microenvironment characteristic of Parkinson’s disease (PD). The central panel depicts a parkinsonian synapse with fragmented collagen IV fibers, degraded perineuronal net (PNN), extracellular α-synuclein aggregates, exosome-mediated propagation, activated microglia, and destabilized postsynaptic GluA1 (AMPA) and integrin β1 (ITGB1) receptors. (**Top left**)**:** Doxycycline, a broad MMP inhibitor with preclinical evidence in PD models. (**Top right**)**:** ATN-161, an integrin β1 antagonist peptide that disrupts focal adhesion kinase (FAK)/Src signaling, with phase 1 evidence in oncology. (**Bottom left**)**:** Mesenchymal stem cells, which secrete tissue inhibitors of metalloproteinases (TIMPs) to restore the MMP–TIMP balance, supported by a randomized clinical trial in PD. (**Bottom right**)**:** Chondroitinase ABC, an experimental approach modulating PNNs around parvalbumin-positive (PV+) interneurons. The level of clinical evidence in PD for each strategy is indicated by the color-coded tags. Created in BioRender. Perez Rubio, R. (2026) https://BioRender.com/d3j8z3s (accessed on 26 May 2026).

ECM disruption also impairs neuronal proteostasis by altering cytoskeletal organization and intracellular trafficking pathways important for protein turnover and aggregate clearance [20]. Aggresome formation requires coordinated interactions between cytoskeletal elements and extracellular adhesion signals; when ECM integrity is lost, these processes are disrupted, promoting the accumulation of misfolded proteins such as α-syn [109,110]. Beyond the ECM+–neuroinflammation–α-syn cycle detailed in Section 6, inflammatory ECM fragments also amplify oxidative stress through TLR-mediated reactive oxygen species production, which in turn damages mitochondrial DNA, proteins, and lipids and compounds nigral neuron vulnerability [60,111,112]. These interconnected mechanisms position the ECM as a convergence point for multiple pathogenic pathways in PD [113]. Variability in extracellular microenvironment stability may explain the phenotypic heterogeneity observed among patients, influencing susceptibility to mitochondrial dysfunction, inflammatory signaling, and synaptic degeneration [11]. This framework may also help explain variable therapeutic responses, such as the suboptimal efficacy of L-DOPA, which could reflect chronic ECM instability that impairs synaptic plasticity and dopaminergic transmission [114,115]. The therapeutic implications of these convergent mechanisms are discussed in Section 8 tand are visually contextualized in Figure 2.

## 8. Therapeutic Implications: MMP Inhibitors and ECM Stabilization Approaches

Targeting the ECM represents a promising therapeutic approach for slowing the progression of PD [17,66]. Dysregulated MMP activity contributes to ECM degradation, and excessive MMP activation has been linked to neuronal injury and synaptic destabilization across neurodegenerative disorders [116]. Pharmacological inhibition of MMPs using repurposed agents such as doxycycline has shown neuroprotective effects in preclinical PD models [67,117]. In MPTP rodent models, doxycycline reduces microglial activation, limits ECM breakdown, and preserves dopaminergic neuron viability in the substantia nigra, with improvement in motor performance in behavioral assays [67]. These findings suggest partial restoration of synaptic architecture and neuronal survival; however, no randomized clinical trial of doxycycline has been completed in PD to date, and its broad-spectrum MMP inhibition together with the microbiome implications of chronic antibiotic exposure constrain straightforward translation. Disruption of ECM–integrin signaling pathways represents another potential therapeutic avenue [20]. Peptide-based inhibitors targeting integrin receptors, such as ATN-161, reduce inflammatory cell migration and microglial activation by blocking ECM–receptor interactions in cellular models [73,74]. ATN-161 has completed a phase 1 trial in patients with solid tumors, where it was well tolerated [73]; its application to PD, however, remains a conceptual extrapolation, with no PD-specific preclinical efficacy data or clinical trials currently available, and CNS penetration in a parkinsonian context has not been characterized. Modulation of PNNs using chondroitinase ABC, a strategy studied in spinal cord injury and stroke models, has been proposed as a conceptual approach to restore synaptic plasticity in PD. Direct evidence in PD models is currently lacking, and the underlying therapeutic question of whether PNNs in PD should be enzymatically digested to restore plasticity or, conversely, stabilized to preserve inhibitory interneuron function, remains conceptually unresolved [22,54]. At present, chondroitinase ABC in PD should be regarded as an experimental concept rather than a translational candidate. Cell-based strategies aimed at stabilizing the extracellular microenvironment are also under investigation. Bone marrow–derived mesenchymal stem cells secrete tissue inhibitors of metalloproteinases and immunomodulatory factors, potentially restoring the MMP–TIMP equilibrium in damaged neural tissue [70,71]. A recent single-center randomized trial of allogeneic bone marrow–derived MSCs in PD reported early safety and tolerability signals together with preliminary efficacy indicators, although the ECM-mediated mechanism of action in humans has not been formally demonstrated, long-term follow-up data remain pending, and multicenter replication is required before broader conclusions can be drawn [72]. The mechanisms of action, supporting evidence, clinical development stage, and key limitations of each of these strategies are detailed in Table 2. Taken together, ECM-targeted strategies in PD currently span four distinct levels of translational maturity: (i) experimental concepts without direct PD data (chondroitinase ABC, selective MMP-3 inhibition); (ii) preclinical evidence in PD-relevant models (doxycycline, MMP-9 inhibition); (iii) clinical-stage agents tested outside PD and proposed for repurposing (ATN-161); and (iv) interventions with early human data in PD (allogeneic mesenchymal stem cells, single randomized trial). No ECM-targeted intervention has yet established disease-modifying efficacy in PD through a confirmatory phase 3 trial. Recognition of these tiers is essential to avoid conflating mechanistic plausibility with clinical readiness.

## 9. Biomarkers and Patient Classification

Biomarkers arising from ECM remodeling represent a useful resource for molecular stratification of PD patients, enabling classification based on underlying pathological mechanisms [118,119]. Proteolytic degradation of structural matrix proteins generates circulating neoepitopes detectable in blood and cerebrospinal fluid, such as C1M and C4M, which show promise as indicators of disease activity and ECM remodeling [120,121]. Elevated levels of these neoepitopes correlate with more aggressive clinical trajectories, including accelerated motor decline and worsening non-motor symptoms [122,123]. The identification of ECM-high progressors—a patient subgroup with persistently elevated ECM biomarker levels and rapid disease progression—offers a basis for patient stratification in clinical trials of ECM-targeted or anti-inflammatory therapies [124]. ECM-derived biomarkers measured in peripheral blood offer practical advantages over more invasive approaches such as lumbar puncture [125]. These biomarkers also capture upstream pathological alterations that precede irreversible neuronal injury, making them useful for early detection and longitudinal monitoring of therapeutic efficacy. Together, ECM-linked biomarkers have potential for advancing personalized medicine and precision care in PD [126].

## 10. Discussion

The multi-omics evidence synthesized in this review supports a central role of the ECM in the synaptic pathophysiology of PD, beyond its traditional function as a passive structural scaffold [17,22,26]. Analyses combining transcriptomic, proteomic, spatial proteomic, and single-cell sequencing datasets show consistent alterations in key ECM components including basement membrane collagens, proteoglycans, and perineuronal network constituents alongside dysregulation of MMPs, TIMPs, and integrin signaling pathways [31,66,85]. These molecular changes affect synaptic stability, dopaminergic neuron vulnerability, and the propagation of α-syn aggregates throughout neural circuits [13,26,108]. This framework connects idiopathic and genetic forms of PD, integrating prior mechanistic models centered on mitochondrial dysfunction, protein aggregation, or neuroinflammation [26,110]. The focus on ECM biology also offers practical translational advantages: ECM-derived biomarkers are accessible in serum or cerebrospinal fluid, supporting early patient stratification and disease monitoring [11]. Consistent validation across experimental platforms including human cohorts, animal models, and cellular systems supports these findings [26,81]. Gene set enrichment analyses across datasets identify dysregulation of pathways governing ECM organization, focal adhesion, and synaptic remodeling, supporting the biological plausibility of ECM dysfunction as a central link between synaptic pathology, neuroinflammation, and clinical progression [20,31]. The ECM offers practical advantages over conventional intracellular targets. Whereas intracellular pathways are often resistant to pharmacological modulation, the ECM is comparatively accessible and pharmacologically tractable [116]. Peripheral stratification using circulating ECM-derived biomarkers, such as collagen neoepitopes, could guide precision targeting of disease-modifying interventions toward those most likely to benefit [118]. Within this framework, inhibition of pathological ECM remodeling represents a testable model for slowing PD progression by preserving synaptic architecture and neuronal microenvironmental stability [17]. Future PD therapeutics will likely involve combinatorial approaches that integrate strategies targeting α-syn aggregation, mitochondrial dysfunction, and neuroinflammatory signaling with ECM stabilization [85,111].

### Limitations

Most of the supporting evidence is correlational; definitive causal studies manipulating specific ECM components in human-relevant models remain scarce [11,26]. Publication bias toward positive ECM findings likely skews the available literature, with few negative or non-confirmatory reports [22]. The neuropathological heterogeneity of PD subtypes introduces confounding variability that has not been systematically controlled across cross-sectional cohorts and complicates the interpretation of group-level ECM signatures [15]. Reports of MMP expression in post-mortem nigral tissue show variable directionality across MMP family members and disease stages [65], and single-cell transcriptomic signatures attributed to ECM-high microglial subpopulations have not been consistently replicated across all published datasets [40,81]. Whether perineuronal net fragmentation precedes or follows dopaminergic loss in early disease also remains mechanistically unresolved [22,38]. Limitations specific to ECM-derived biomarkers: The translational case for C1M and C4M as prognostic biomarkers in PD rests on a small number of cohorts, notably ICICLE-PD, and lacks prospective multicenter validation [41,84]. These neoepitopes are not central nervous system–specific: collagen turnover from peripheral tissues, including liver, kidney, and vasculature, contributes to circulating levels, raising concerns about specificity in older patients with frequent systemic comorbidities [49,121]. Reference ranges, assay standardization, and clinically actionable cut-points are not yet established. Comparative performance against neurofilament light chain rests on limited head-to-head data, and definitive superiority of ECM-derived neoepitopes over NfL cannot be claimed at present [79,82]. No ECM-based biomarker has been validated for differential diagnosis between PD and atypical parkinsonian syndromes. Limitations of ECM-targeted therapeutic strategies. As detailed in Table 2, no ECM-targeted intervention has completed a confirmatory randomized clinical trial in PD, except for a single-center preliminary mesenchymal stem cell study [72]. Central nervous system penetration, molecular target selectivity, and pharmacodynamic response biomarkers remain unresolved for most candidate agents. Whether ECM modulation should favor stabilization or controlled degradation of specific structures, such as perineuronal nets, also remains conceptually unresolved.

## 11. Concluding Remarks and Future Directions

The evidence synthesized in this review supports ECM dysregulation as an active contributor to PD pathogenesis, linking synaptic dysfunction, neuroinflammation, and prion-like α-syn propagation [17,85]. Remodeling of structural ECM constituents—including basement membrane collagens, proteoglycans, and PNNs— shapes the neuronal microenvironment that supports synaptic integrity and dopaminergic resilience and influences the susceptibility of neural circuits to mitochondrial dysfunction, glial activation, and proteopathic spread [19,20,85,107]. The relative accessibility of the ECM, compared with intracellular targets, and the availability of peripheral ECM-derived biomarkers, such as collagen neoepitopes detectable in blood, offer a practical path from mechanistic insight to clinical application, with utility in early patient stratification, longitudinal disease monitoring, and biomarker-guided trial design [49,118,119,125]. Therapeutic strategies targeting matrix MMP activity, integrin signaling, and ECM turnover represent reasonable candidates for preserving synaptic architecture and neuronal viability; clinical translation will require in vivo validation and international collaboration [17,26,116,118]. Future investigations should employ experimental systems that recapitulate the structural and molecular complexity of the human brain [26,127]. iPSC-derived midbrain organoids integrated with three-dimensional bioprinted scaffolds incorporating collagen IV, aggrecan, and perlecan may allow more faithful reconstruction of disease-relevant microenvironments [22,54]. Combining these platforms with high-throughput multi-omics, spatial proteomics, and single-cell chromatin accessibility profiling can provide longitudinal data on ECM remodeling across PD progression [26,111]. Large-scale, standardized datasets analyzed with artificial intelligence and machine learning may help identify optimal therapeutic windows and biomarker signatures [118]. Genome-editing technologies such as CRISPR–Cas9 may enable targeted modulation of ECM-related genes, potentially preserving perineuronal nets and synaptic architecture in vivo [17,104,116]. Achieving these goals will require global consortia, standardized biobanking, and coordinated research initiatives [26,118]. By integrating bioengineering, multi-omics, computational analytics, and precision medicine, PD may be reframed as a stratifiable and modifiable disorder in which ECM represents a biologically relevant and therapeutically tractable axis [85,118].

## Figures and Tables

**Table 1:** Summary matrix of extracellular matrix components dysregulated in Parkinson’s disease: alterations, mechanistic consequences, evidence base, and translational implications.

ECM Component	Alteration in PD	Cellular/Synaptic Consequence	Type of Evidence	Translational Implication	Evidence Strength	Key References
Collagen IV (COL4A1, COL4A2)	Downregulation	Destabilization of basement membrane scaffold; aberrant presynaptic vesicle docking; proposed indirect impairment of PINK1/Parkin-mediated mitophagy via integrin–cytoskeletal coupling (working model)	Bulk RNA-seq of human SN; iPSC-derived DA neurons	Candidate biomarker (C4M neoepitope) and target for ECM stabilization	High	[26,31,36,37,42,53]
Collagen VI (COL6A1, COL6A2)	Downregulation	Loss of perineuronal scaffolding; reduced neuroprotection against oxidative stress	Bulk and single-cell transcriptomics in SN and cortex	Indirect biomarker; under-explored therapeutically	Moderate	[31,35,40]
Aggrecan (ACAN)	Decreased; PNN fragmentation	Loss of PNN integrity around PV+ interneurons; impaired plasticity, vulnerability of inhibitory tone	Post-mortem human tissue (hippocampus, PFC); A53T transgenic mice	Potential synaptic-resilience target	High	[17,22,38,39,54]
Hyaluronan	Fragmentation (low-MW HA accumulation)	Acts as DAMP; activates TLR2/TLR4 on microglia and astrocytes (A1 phenotype)	MPTP rodent models; in vitro glial cultures	Anti-inflammatory targeting via TLR pathway	Moderate	[55,56,57,58]
Biglycan	Increased extracellular release	TLR2/TLR4 activation; NF-κB and MAPK signaling; cytokine release	Mechanistic in vitro studies; extrapolated to PD context	Indirect target via TLR modulation	Low	[59,60,61]
Tenascin-C	Upregulation in neuroinflammatory states	Sustained neuroinflammation; modulation of synaptic remodeling	Reviewed mechanistic evidence in CNS disorders	Emerging anti-inflammatory target	Low	[62]
MMP-9	Upregulation	Degradation of basement membrane collagens and PNN; BBB disruption; promotes prion-like α-syn spread	Post-mortem PD tissue; MPTP and A53T models; genetic KO restores DA neurons	Pharmacological inhibition (e.g., doxycycline)—preclinical neuroprotection	High	[63,64,65,66,67]
MMP-3	Upregulation; intracellular pro-apoptotic role	Contributes to nigrostriatal DA neuronal death and BBB damage	MPTP mouse model; in vitro DA cell apoptosis	Selective MMP-3 inhibition—preclinical	Moderate	[68,69]
MMP-13, MMP-14	Upregulation	ECM remodeling; generation of collagen neoepitopes (C1M, C4M)	Bulk transcriptomics; serum ELISA neoepitope assays	Source of circulating biomarkers	Moderate	[31,41,49]
TIMP1, TIMP3	Dysregulated expression (imbalance with MMPs)	Loss of MMP–TIMP equilibrium; sustained ECM degradation	Bulk transcriptomics; post-mortem tissue	Restoration via MSC-secreted TIMPs (preclinical)	Moderate	[31,65,70,71,72]
Integrin β1 (ITGB1)	Downregulation	Disruption of FAK/Src signaling; impaired CaMKII-dependent LTP and GluA1 anchoring; postsynaptic destabilization	scRNA-seq of iPSC-DA neurons; cellular models	Integrin-stabilizing peptides (e.g., ATN-161)— preclinical	Moderate	[36,37,46,47,73,74]
Vinculin (VCL)	Downregulation	Weakened focal adhesion complexes; reduced synaptic anchoring; dendritic simplification	scRNA-seq of iPSC-DA neurons	Indirect target via integrin axis	Low	[36,37,47]

Systematic synthesis of extracellular matrix (ECM) components with altered expression or function in Parkinson’s disease, organized by molecular class (structural collagens, proteoglycans, glycosaminoglycans, matrix metalloproteinases and their inhibitors, and integrins). The heterogeneity in evidence types reflects the nascent state of the field and underscores the need for longitudinal mechanistic studies integrating human models with in vivo validation. **Note:** For evidence strength, High = consistent findings across human tissue, animal models and independent cohorts; Moderate = supported by one or two orthogonal lines of evidence or predominantly in vitro data; Low = mainly speculative, extrapolated from other CNS disorders, or based on single exploratory datasets. Abbreviations: BBB, blood–brain barrier; CaMKII, Ca^2+^/calmodulin-dependent protein kinase II; CSPG, chondroitin sulfate proteoglycan; DA, dopaminergic; DAMP, damage-associated molecular pattern; ECM, extracellular matrix; FAK, focal adhesion kinase; HA, hyaluronan; iPSC, induced pluripotent stem cell; KO, knockout; LTP, long-term potentiation; MMP, matrix metalloproteinase; MW, molecular weight; PD, Parkinson’s disease; PFC, prefrontal cortex; PNN, perineuronal net; PV+, parvalbumin-positive; scRNA-seq, single-cell RNA sequencing; *SN*, *substantia nigra*; TH, tyrosine hydroxylase; TIMP, tissue inhibitor of metalloproteinase; TLR, Toll-like receptor.

**Table 2:** Extracellular matrix-targeted therapeutic strategies in Parkinson’s disease: mechanism, evidence, and limitations.

Strategy/Agent	Mechanism of Action	Preclinical Evidence (PD- Relevant)	Clinical Evidence in PD	Limitations/ Caveats
Doxycycline (broad MMP inhibitor)	Inhibits MMP activity; suppresses microglial p38 MAPK and NF-κB; modulates α-syn aggregation	MPTP mouse: reduced microglial activation, preserved DA neurons, improved motor performance [67]. In vitro/in vivo: inhibits α-syn-associated pathology [117]	No randomized controlled trial in PD to date (as of 2025)	Broad-spectrum effect (not ECM-specific); long-term antibiotic use raises microbiome concerns; clinical efficacy uncertain
Selective MMP-9 inhibition (genetic/pharmacological)	Blocks MMP-9-driven ECM degradation, BBB disruption, and neuroinflammation	Genetic ablation or pharmacological MMP-9 inhibition partially restores TH+ neurons in α-syn transgenic models [64,65]	No clinical PD trials. Past broad MMP inhibitors (e.g., marimastat) failed in oncology due to musculoskeletal toxicity	Selectivity for MMP-9 vs. other MMPs is technically challenging; off-target risk
Selective MMP-3 inhibition	Blocks both extracellular ECM degradation and intracellular pro-apoptotic role of MMP-3	MPTP mouse: reduced nigrostriatal DA loss, BBB protection [68,69]	No clinical PD trials	Early-stage; selective inhibitors not yet clinically available for CNS use
ATN-161 (integrin β1 antagonist peptide)	Blocks ECM–integrin interactions; reduces inflammatory cell migration and microglial activation	Cellular models: attenuates pro-inflammatory cytokine production [73,74]	Phase 1 in solid tumors completed [73]; no PD trials	Repurposing to PD remains hypothetical; CNS penetration not characterized for PD context
Allogeneic bone marrow-derived MSCs	Secretion of TIMPs; restoration of MMP–TIMP equilibrium; immunomodulation	Animal models: enhanced neuronal survival, reduced neuroinflammation [70,71]	Randomized trial in PD reported (Schiess et al., 2025)—early efficacy and safety signals [72]	Single-center; limited long-term follow-up; mechanism in humans not formally demonstrated to be ECM-mediated
PNN modulation (chondroitinase ABC and analogues)	Enzymatic digestion of CSPGs to restore plasticity; conceptually opposite to PNN stabilization	Mostly tested in spinal cord injury and stroke; PD-specific data limited	No PD clinical trials	Therapeutic direction (digest vs. stabilize PNN) in PD remains conceptually unresolved

Six strategies are compared across four domains: mechanism of action, preclinical evidence in PD-relevant models, available clinical evidence, and key limitations or caveats. Strategies targeting matrix metalloproteinases—including broad inhibition with doxycycline and selective inhibition of MMP-3 and MMP-9—aim to reduce ECM degradation, BBB disruption, and neuroinflammation in dopaminergic nigrostriatal circuits, with supporting evidence drawn primarily from MPTP mouse models and TH-positive neuron quantification in alpha-synuclein transgenic lines; no randomized controlled trials in PD have been conducted for any of these agents to date. Integrin-targeted therapy with ATN-161 and allogeneic MSC transplantation—the latter acting through TIMP secretion to restore MMP–TIMP equilibrium—represent biologics-based approaches with preliminary or early-phase clinical data. PNN modulation via chondroitinase ABC-mediated CSPG digestion remains at the conceptual stage for PD, with the therapeutic direction unresolved. CNS penetration, target selectivity, and the absence of PD-specific clinical trials constitute shared limitations across most strategies reviewed. Abbreviations: BBB, blood–brain barrier; CNS, central nervous system; CSPG, chondroitin sulfate proteoglycan; DA, dopaminergic; ECM, extracellular matrix; MMP, matrix metalloproteinase; MPTP, 1-methyl-4-phenyl-1,2,3,6-tetrahydropyridine; MSC, mesenchymal stem cell; PD, Parkinson’s disease; PNN, perineuronal net; TH, tyrosine hydroxylase; TIMP, tissue inhibitor of metalloproteinase.

## Data Availability

Not applicable. This article is a narrative review and did not generate new data.

## Abbreviations

The following abbreviations are used in this manuscript:

α-syn	Alpha-synuclein
ACAN	Aggrecan
AMPA	α-amino-3-hydroxy-5-methyl-4-isoxazolepropionic acid
ATN-161	Ac-PHSCN-NH2 (integrin β1 antagonist peptide)
BBB	Blood–brain barrier
CaMKII	Ca^2+^/calmodulin-dependent protein kinase II
CNS	Central nervous system
COL4A1/A2	Collagen type IV, alpha 1/alpha 2
COL6A1/A2	Collagen type VI, alpha 1/alpha 2
CRISPR–Cas9	Clustered regularly interspaced short palindromic repeats/CRISPR-associated protein 9
CSF	Cerebrospinal fluid
CSPG	Chondroitin sulfate proteoglycan
C1M	MMP-generated type I collagen neoepitope
C4M	MMP-generated type IV collagen neoepitope
DA	Dopaminergic
DAMP	Damage-associated molecular pattern
ECM	Extracellular matrix
ELISA	Enzyme-linked immunosorbent assay
FAK	Focal adhesion kinase
GBA	Glucocerebrosidase
GluA1	Glutamate receptor AMPA subunit 1
GSEA	Gene set enrichment analysis
GTPase	Guanosine triphosphatase
HA	Hyaluronan
ICICLE-PD	Incidence of Cognitive Impairment in Cohorts with Longitudinal Evaluation in Parkinson’s Disease
IL-1β	Interleukin 1 beta
IL-6	Interleukin 6
iPSC	Induced pluripotent stem cell
ITGB1	Integrin beta 1
KO	Knockout
L-DOPA	Levodopa
LC-MS/MS	Liquid chromatography–tandem mass spectrometry
LRRK2	Leucine-rich repeat kinase 2
LTP	Long-term potentiation
MAPK	Mitogen-activated protein kinase
MMP	Matrix metalloproteinase
MPTP	1-methyl-4-phenyl-1,2,3,6-tetrahydropyridine
MSC	Mesenchymal stem cell
MW	Molecular weight
NF-κB	Nuclear factor kappa B
NfL	Neurofilament light chain
NINDS	National Institute of Neurological Disorders and Stroke
PD	Parkinson’s disease
PFC	Prefrontal cortex
PINK1	PTEN-induced kinase 1
PNN	Perineuronal net
PPMI	Parkinson Progression Marker Initiative
PRKN	Parkin RBR E3 ubiquitin protein ligase
PSD95	Postsynaptic density protein 95
PV+	Parvalbumin-positive
Rab	Ras-related protein
RNA-seq	RNA sequencing
ROS	Reactive oxygen species
scRNA-seq	Single-cell RNA sequencing
Ser129	Serine 129
*SNpc*	*Substantia nigra pars compacta*
SNCA	Synuclein alpha gene
Src	Proto-oncogene tyrosine-protein kinase Src
TH	Tyrosine hydroxylase
TIMP	Tissue inhibitor of metalloproteinase
TLR2	Toll-like receptor 2
TLR4	Toll-like receptor 4
TNF-α	Tumor necrosis factor alpha
UPDRS	Unified Parkinson’s Disease Rating Scale
VCL	Vinculin
VDAC	Voltage-dependent anion channel

## References

[1] Dorsey E.R., Sherer T., Okun M.S., Bloem B.R. (2018). The Emerging Evidence of the Parkinson Pandemic. J. Park. Dis..

[2] World Health Organization (2023). Parkinson Disease. https://www.who.int/news-room/fact-sheets/detail/parkinson-disease.

[3] National Institute of Environmental Health Sciences (NIEHS) (2025). Parkinson’s Disease. https://www.niehs.nih.gov/health/topics/conditions/parkinson.

[4] Poewe W., Seppi K., Tanner C.M., Halliday G.M., Brundin P., Volkmann J., Schrag A.E., Lang A.E. (2017). Parkinson disease. Nat. Rev. Dis. Primers.

[5] Song J., Kim J. (2016). Degeneration of dopaminergic neurons due to metabolic alterations and Parkinson’s disease. Front. Aging Neurosci..

[6] Dauer W., Przedborski S. (2003). Parkinson’s disease: Mechanisms and models. Neuron.

[7] Chaudhuri K.R., Healy D.G., Schapira A.H., National Institute for Clinical Excellence (2006). Non-motor symptoms of Parkinson’s disease: Diagnosis and management. Lancet Neurol..

[8] Spillantini M.G., Schmidt M.L., Lee V.M., Trojanowski J.Q., Jakes R., Goedert M. (1997). Alpha-synuclein in Lewy bodies. Nature.

[9] Hirsch E.C., Hunot S. (2009). Neuroinflammation in Parkinson’s disease: A target for neuroprotection?. Lancet Neurol..

[10] Exner N., Lutz A.K., Haass C., Winklhofer K.F. (2012). Mitochondrial dysfunction in Parkinson’s disease: Molecular mechanisms and pathophysiological consequences. EMBO J..

[11] Surmeier D.J., Obeso J.A., Halliday G.M. (2017). Selective neuronal vulnerability in Parkinson disease. Nat. Rev. Neurosci..

[12] Schapira A.H.V., Chaudhuri K.R., Jenner P. (2017). Non-motor features of Parkinson disease. Nat. Rev. Neurosci..

[13] Brundin P., Dave K.D., Kordower J.H. (2017). Therapeutic approaches to target alpha-synuclein pathology. Exp. Neurol..

[14] Brundin P., Melki R., Kopito R. (2010). Prion-like transmission of protein aggregates in neurodegenerative diseases. Nat. Rev. Mol. Cell Biol..

[15] Braak H., Del Tredici K., Rüb U., de Vos R.A., Jansen Steur E.N., Braak E. (2003). Staging of brain pathology related to sporadic Parkinson’s disease. Neurobiol. Aging.

[16] Kordower J.H., Chu Y., Hauser R.A., Freeman T.B., Olanow C.W. (2008). Lewy body-like pathology in long-term embryonic nigral transplants in Parkinson’s disease. Nat. Med..

[17] Dityatev A., Schachner M. (2003). Extracellular matrix molecules and synaptic plasticity. Nat. Rev. Neurosci..

[18] Frischknecht R., Gundelfinger E.D. (2012). The brain’s extracellular matrix and its role in synaptic plasticity. Adv. Exp. Med. Biol..

[19] Frantz C., Stewart K.M., Weaver V.M. (2010). The extracellular matrix at a glance. J. Cell Sci..

[20] Hynes R.O. (2009). The extracellular matrix: Not just pretty fibrils. Science.

[21] Chelyshev Y.A., Kabdesh I.M., Mukhamedshina Y.O. (2022). Extracellular Matrix in Neural Plasticity and Regeneration. Cell. Mol. Neurobiol..

[22] Sorg B.A., Berretta S., Blacktop J.M., Fawcett J.W., Kitagawa H., Kwok J.C.F., Miquel M. (2016). Casting a Wide Net: Role of Perineuronal Nets in Neural Plasticity. J. Neurosci..

[23] Zhang X., Ma Y., Gao G., Wu Q. (2025). Aggregation and Propagation of α-Synuclein in Parkinson’s Disease: A Bibliometric Perspective. Brain Behav..

[24] Fawcett J.W., Oohashi T., Pizzorusso T. (2019). The roles of perineuronal nets and the perinodal extracellular matrix in neuronal function. Nat. Rev. Neurosci..

[25] Blauwendraat C., Nalls M.A., Singleton A.B. (2020). The genetic architecture of Parkinson’s disease. Lancet Neurol..

[26] Dumitriu A., Golji J., Labadorf A.T., Gao B., Beach T.G., Myers R.H., Longo K.A., Latourelle J.C. (2016). Integrative analyses of proteomics and RNA transcriptomics implicate mitochondrial processes, protein folding pathways and GWAS loci in Parkinson disease. BMC Med. Genom..

[27] Lin L., Göke J., Cukuroglu E., Dranias M.R., VanDongen A.M., Stanton L.W. (2016). Molecular Features Underlying Neurodegeneration Identified through In Vitro Modeling of Genetically Diverse Parkinson’s Disease Patients. Cell Rep..

[28] Johnson E.C.B., Dammer E.B., Duong D.M., Ping L., Zhou M., Yin L., Higginbotham L., Guajardo A., White B., Troncoso J.C. (2020). Large-scale proteomic analysis of Alzheimer’s disease brain and cerebrospinal fluid reveals early changes in energy metabolism associated with microglia and astrocyte activation. Nat. Med..

[29] Wang Q., Liu Y., Zhou J. (2015). Neuroinflammation in Parkinson’s disease and its potential as therapeutic target. Transl. Neurodegener..

[30] Rike W.A., Stern S. (2023). Proteins and Transcriptional Dysregulation of the Brain Extracellular Matrix in Parkinson’s Disease: A Systematic Review. Int. J. Mol. Sci..

[31] Rivera S., García-González L., Khrestchatisky M., Baranger K. (2019). Metalloproteinases and their tissue inhibitors in Alzheimer’s disease and other neurodegenerative disorders. Cell Mol. Life Sci..

[32] Volpicelli-Daley L.A., Luk K.C., Patel T.P., Tanik S.A., Riddle D.M., Stieber A., Meaney D.F., Trojanowski J.Q., Lee V.M. (2011). Exogenous α-synuclein fibrils induce Lewy body pathology leading to synaptic dysfunction and neuron death. Neuron.

[33] Magdalinou N.K., Lees A.J., Zetterberg H. (2014). Cerebrospinal fluid biomarkers in parkinsonian conditions: An update and future directions. J. Neurol. Neurosurg. Psychiatry.

[34] Liu W., Xu L., Wang X., Wang J. (2026). Integrative multi-omics and network-based machine learning for early diagnosis of Parkinson’s disease. PLoS ONE.

[35] Chapman M.A., Sorg B.A. (2024). A Systematic Review of Extracellular Matrix-Related Alterations in Parkinson’s Disease. Brain Sci..

[36] Rosh I., Tripathi U., Hussein Y., Rike W.A., Djamus J., Shklyar B., Manole A., Houlden H., Winkler J., Gage F.H. (2024). Synaptic dysfunction and extracellular matrix dysregulation in dopaminergic neurons from sporadic and E326K-GBA1 Parkinson’s disease patients. npj Park. Dis..

[37] Stern S., Lau S., Manole A., Rosh I., Percia M.M., Ben Ezer R., Shokhirev M.N., Qiu F., Schafer S., Mansour A.A. (2022). Reduced synaptic activity and dysregulated extracellular matrix pathways in midbrain neurons from Parkinson’s disease patients. npj Park. Dis..

[38] Oaks A.W., Frankfurt M., Finkelstein D.I., Sidhu A. (2013). Age-dependent effects of A53T alpha-synuclein on behavior and dopaminergic function. PLoS ONE.

[39] Morawski M., Brückner M.K., Riederer P., Brückner G., Arendt T. (2004). Perineuronal nets potentially protect against oxidative stress. Exp. Neurol..

[40] Dijkstra A.A., Ingrassia A., de Menezes R.X., van Kesteren R.E., Rozemuller A.J.M., Heutink P., van de Berg W.D.J. (2015). Evidence for Immune Response, Axonal Dysfunction and Reduced Endocytosis in the Substantia Nigra in Early Stage Parkinson’s Disease. PLoS ONE.

[41] Holm Nielsen S., Karsdal M., Henriksen K. (2025). Diagnostic value of extracellular matrix degradation biomarkers in serum from patients with Parkinson’s disease. J. Neurol. Sci..

[42] Thomsen M.S., Routhe L.J., Moos T. (2017). The vascular basement membrane in the healthy and pathological brain. J. Cereb. Blood Flow Metab..

[43] Zhang Y., James M., Middleton F.A., Davis R.L. (2005). Transcriptional analysis of multiple brain regions in Parkinson’s disease supports the involvement of specific protein processing, energy metabolism, and signaling pathways, and suggests novel disease mechanisms. Am. J. Med. Genet B Neuropsychiatr. Genet..

[44] Szklarczyk D., Gable A.L., Nastou K.C., Lyon D., Kirsch R., Pyysalo S., Doncheva N.T., Legeay M., Fang T., Bork P. (2021). The STRING database in 2021: Customizable protein-protein networks, and functional characterization of user-uploaded gene/measurement sets. Nucleic Acids Res..

[45] Ryan S.D., Dolatabadi N., Chan S.F., Zhang X., Akhtar M.W., Parker J., Soldner F., Sunico C.R., Nagar S., Talantova M. (2013). Isogenic human iPSC Parkinson’s model shows nitrosative stress-induced dysfunction in MEF2-PGC1α transcription. Cell..

[46] Luo P., Yang Y., Liu W., Rao W., Bian H., Li X., Chen T., Liu M., Zhao Y., Dai S. (2013). Downregulation of postsynaptic density-95-interacting regulator of spine morphogenesis reduces glutamate-induced excitotoxicity by differentially regulating glutamate receptors in rat cortical neurons. FEBS J..

[47] Lilja J., Ivaska J. (2018). Integrin activity in neuronal connectivity. J. Cell Sci..

[48] Parnetti L., Gaetani L., Eusebi P., Paciotti S., Hansson O., El-Agnaf O., Mollenhauer B., Blennow K., Calabresi P. (2019). CSF and blood biomarkers for Parkinson’s disease. Lancet Neurol..

[49] Leeming D.J., He Y., Veidal S.S., Nguyen Q.H., Larsen D.V., Koizumi M., Segovia-Silvestre T., Zhang C., Zheng Q., Sun S. (2011). A novel marker for assessment of liver matrix remodeling: An enzyme-linked immunosorbent assay (ELISA) detecting a MMP generated type I collagen neo-epitope (C1M). Biomarkers.

[50] Fujiwara H., Hasegawa M., Dohmae N., Kawashima A., Masliah E., Goldberg M.S., Shen J., Takio K., Iwatsubo T. (2002). α-Synuclein is phosphorylated in synucleinopathy lesions. Nat. Cell Biol..

[51] Anderson J.P., Walker D.E., Goldstein J.M., de Laat R., Banducci K., Caccavello R.J., Barbour R., Huang J., Kling K., Lee M. (2006). Phosphorylation of Ser-129 is the dominant pathological modification of alpha-synuclein in familial and sporadic Lewy body disease. J. Biol. Chem..

[52] Tokuda T., Salem S.A., Allsop D., Mizuno T., Nakagawa M., Qureshi M.M., Locascio J.J., Schlossmacher M.G., El-Agnaf O.M. (2006). Decreased alpha-synuclein in cerebrospinal fluid of aged individuals and subjects with Parkinson’s disease. Biochem. Biophys. Res. Commun..

[53] Narendra D., Tanaka A., Suen D.F., Youle R.J. (2008). Parkin is recruited selectively to impaired mitochondria and promotes their autophagy. J. Cell Biol..

[54] Soleman S., Filippov M.A., Dityatev A., Fawcett J.W. (2013). Targeting the neural extracellular matrix in neurological disorders. Neuroscience.

[55] Meredith G.E., Rademacher D.J. (2011). MPTP mouse models of Parkinson’s disease: An update. J. Park. Dis..

[56] Lawton M., Baig F., Rolinski M., Ruffman C., Nithi K., May M.T., Ben-Shlomo Y., Hu M.T. (2015). Parkinson’s Disease Subtypes in the Oxford Parkinson Disease Centre (OPDC) Discovery Cohort. J. Park. Dis..

[57] Shastri A., Bonifati D.M., Kishore U. (2013). Innate immunity and neuroinflammation. Mediat. Inflamm..

[58] Jiang D., Liang J., Noble P.W. (2007). Hyaluronan in tissue injury and repair. Annu. Rev. Cell Dev. Biol..

[59] Schaefer L., Babelova A., Kiss E., Hausser H.J., Baliova M., Krzyzankova M., Marsche G., Young M.F., Mihalik D., Götte M. (2005). The matrix component biglycan is proinflammatory and signals through Toll-like receptors 4 and 2 in macrophages. J. Clin. Investig..

[60] Kawai T., Akira S. (2010). The role of pattern-recognition receptors in innate immunity: Update on Toll-like receptors. Nat. Immunol..

[61] Takeda K., Akira S. (2005). Toll-like receptors in innate immunity. Int. Immunol..

[62] Jin Y.L., Bao S.W., Huang M.X., Gao Y.J., Lu H.J., Wu X.B. (2025). The Role of Tenascin-C in Neuroinflammation and Neuroplasticity. Int. J. Mol. Sci..

[63] Annese V., Herrero M.T., Di Pentima M., Gomez A., Lombardi L., Ros C.M., De Pablos V., Fernandez-Villalba E., De Stefano M.E. (2015). Metalloproteinase-9 contributes to inflammatory glia activation and nigro-striatal pathway degeneration in both mouse and monkey models of 1-methyl-4-phenyl-1,2,3,6-tetrahydropyridine (MPTP)-induced Parkinsonism. Brain Struct. Funct..

[64] Rosenberg G.A. (2009). Matrix metalloproteinases and their multiple roles in neurodegenerative diseases. Lancet Neurol..

[65] Lorenzl S., Albers D.S., Narr S., Chirichigno J., Beal M.F. (2002). Expression of MMP-2, MMP-9, and MMP-1 and Their Endogenous Counterregulators TIMP-1 and TIMP-2 in Postmortem Brain Tissue of Parkinson’s Disease. Exp. Neurol..

[66] Rempe R.G., Hartz A.M.S., Bauer B. (2016). Matrix metalloproteinases in the brain and blood-brain barrier: Versatile breakers and makers. J. Cereb. Blood Flow Metab..

[67] Santa-Cecília F.V., Socias B., Ouidja M.O., Sepulveda-Diaz J.E., Acuña L., Silva R.L., Michel P.P., Del-Bel E., Cunha T.M., Raisman-Vozari R. (2016). Doxycycline Suppresses Microglial Activation by Inhibiting the p38 MAPK and NF-kB Signaling Pathways. Neurotox. Res..

[68] Chung Y.C., Kim Y.S., Bok E., Yune T.Y., Maeng S., Jin B.K. (2013). MMP-3 contributes to nigrostriatal dopaminergic neuronal loss, BBB damage, and neuroinflammation in an MPTP mouse model of Parkinson’s disease. Mediat. Inflamm..

[69] Choi D.H., Kim E.M., Son H.J., Joh T.H., Kim Y.S., Kim D., Flint Beal M., Hwang O. (2008). A novel intracellular role of matrix metalloproteinase-3 during apoptosis of dopaminergic cells. J. Neurochem..

[70] Uccelli A., Moretta L., Pistoia V. (2008). Mesenchymal stem cells in health and disease. Nat. Rev. Immunol..

[71] Caplan A.I., Dennis J.E. (2006). Mesenchymal stem cells as trophic mediators. J. Cell. Biochem..

[72] Schiess M.C., Suescun J., Martinez-Lemus J.D., Green C., Thomas T.S., Shahnawaz M., Tharp E., Satani N.B., Saltarrelli J.G., Adams C. (2025). Allogeneic Bone Marrow-Derived Mesenchymal Stem Cells for Parkinson’s Disease: A Randomized Trial. Mov. Disord..

[73] Cianfrocca M.E., Kimmel K.A., Gallo J., Cardoso T., Brown M.M., Hudes G., Lewis N., Weiner L., Lam G.N., Brown S.C. (2006). Phase 1 trial of the antiangiogenic peptide ATN-161 (Ac-PHSCN-NH2), a beta integrin antagonist, in patients with solid tumours. Br. J. Cancer.

[74] Harburger D.S., Calderwood D.A. (2009). Integrin signalling at a glance. J. Cell Sci..

[75] Domingo-Lopez D.A., de Armas M.R.A., Martin-Saldaña S. (2026). Extracellular matrix remodeling therapeutic strategies to tackle central nervous system diseases. Neural Regen. Res..

[76] Bagetta V., Ghiglieri V., Sgobio C., Calabresi P., Picconi B. (2010). Synaptic dysfunction in Parkinson’s disease. Biochem. Soc. Trans..

[77] Desplats P., Lee H.J., Bae E.J., Patrick C., Rockenstein E., Crews L., Spencer B., Masliah E., Lee S.J. (2009). Inclusion formation and neuronal cell death through neuron-to-neuron transmission of alpha-synuclein. Proc. Natl. Acad. Sci. USA.

[78] Liddelow S.A., Guttenplan K.A., Clarke L.E., Bennett F.C., Bohlen C.J., Schirmer L., Bennett M.L., Münch A.E., Chung W.S., Peterson T.C. (2017). Neurotoxic reactive astrocytes are induced by activated microglia. Nature.

[79] Hansson O., Janelidze S., Hall S., Magdalinou N., Lees A.J., Andreasson U., Norgren N., Linder J., Forsgren L., Constantinescu R. (2017). Blood-based NfL: A biomarker for differential diagnosis of parkinsonian disorder. Neurology.

[80] Marek K., Jennings D., Lasch S., Siderowf A., Tanner C., Simuni T., Coffey C., Kieburtz K., Flagg E., Chowdhury S. (2011). The Parkinson Progression Marker Initiative (PPMI). Prog. Neurobiol..

[81] Zhu B., Park J.M., Coffey S.R., Russo A., Hsu I.U., Wang J., Su C., Chang R., Lam T.T., Gopal P.P. (2024). Single-cell transcriptomic and proteomic analysis of Parkinson’s disease brains. Sci. Transl. Med..

[82] Mollenhauer B., Dakna M., Kruse N., Galasko D., Foroud T., Zetterberg H., Schade S., Gera R.G., Wang W., Gao F. (2020). Validation of Serum Neurofilament Light Chain as a Biomarker of Parkinson’s Disease Progression. Mov. Disord..

[83] Aamodt W.W., Waligorska T., Shen J., Tropea T.F., Siderowf A., Weintraub D., Grossman M., Irwin D., Wolk D.A., Xie S.X. (2021). Neurofilament Light Chain as a Biomarker for Cognitive Decline in Parkinson Disease. Mov. Disord..

[84] Yarnall A.J., Breen D.P., Duncan G.W., Khoo T.K., Coleman S.Y., Firbank M.J., Nombela C., Winder-Rhodes S., Evans J., Rowe J.B. (2014). Characterizing mild cognitive impairment in incident Parkinson disease: The ICICLE-PD study. Neurology.

[85] Guo J.L., Lee V.M. (2014). Cell-to-cell transmission of pathogenic proteins in neurodegenerative diseases. Nat. Med..

[86] Mollenhauer B., Locascio J.J., Schulz-Schaeffer W., Sixel-Döring F., Trenkwalder C., Schlossmacher M.G. (2011). α-Synuclein and tau concentrations in cerebrospinal fluid of patients presenting with parkinsonism: A cohort study. Lancet Neurol..

[87] Fereshtehnejad S.M., Zeighami Y., Dagher A., Postuma R.B. (2017). Clinical criteria for subtyping Parkinson’s disease: Biomarkers and longitudinal progression. Brain.

[88] Lisman J., Yasuda R., Raghavachari S. (2012). Mechanisms of CaMKII action in long-term potentiation. Nat. Rev. Neurosci..

[89] Malenka R.C., Bear M.F. (2004). LTP and LTD: An embarrassment of riches. Neuron.

[90] Shepherd J.D., Huganir R.L. (2007). The cell biology of synaptic plasticity: AMPA receptor trafficking. Annu. Rev. Cell Dev. Biol..

[91] Lee H.K., Takamiya K., Han J.S., Man H., Kim C.H., Rumbaugh G., Yu S., Ding L., He C., Petralia R.S. (2003). Phosphorylation of the AMPA receptor GluR1 subunit is required for synaptic plasticity and retention of spatial memory. Cell.

[92] Impey S., Obrietan K., Storm D.R. (1999). Making new connections: Role of ERK/MAP kinase signaling in neuronal plasticity. Neuron.

[93] Brunet A., Datta S.R., Greenberg M.E. (2001). Transcription-dependent and -independent control of neuronal survival by the PI3K-Akt signaling pathway. Curr. Opin. Neurobiol..

[94] Emmanouilidou E., Melachroinou K., Roumeliotis T., Garbis S.D., Ntzouni M., Margaritis L.H., Stefanis L., Vekrellis K. (2010). Cell-produced alpha-synuclein is secreted in a calcium-dependent manner by exosomes and impacts neuronal survival. J. Neurosci..

[95] Danzer K.M., Kranich L.R., Ruf W.P., Cagsal-Getkin O., Winslow A.R., Zhu L., Vanderburg C.R., McLean P.J. (2012). Exosomal cell-to-cell transmission of alpha synuclein oligomers. Mol. Neurodegener..

[96] Dieriks B.V., Park T.I., Fourie C., Faull R.L., Dragunow M., Curtis M.A. (2017). α-synuclein transfer through tunneling nanotubes occurs in SH-SY5Y cells and primary brain pericytes from Parkinson’s disease patients. Sci. Rep..

[97] Abounit S., Wu J.W., Duff K., Victoria G.S., Zurzolo C. (2016). Tunneling nanotubes: A possible highway in the spreading of tau and other prion-like proteins in neurodegenerative diseases. Prion.

[98] Rostami J., Holmqvist S., Lindström V., Sigvardson J., Westermark G.T., Ingelsson M., Bergström J., Roybon L., Erlandsson A. (2017). Human astrocytes transfer aggregated alpha-synuclein via tunneling nanotubes. J. Neurosci..

[99] Zindel J., Kubes P. (2020). DAMPs, PAMPs, and LAMPs in Immunity and Sterile Inflammation. Annu. Rev. Pathol..

[100] Lehnardt S. (2010). Innate immunity and neuroinflammation in the CNS: The role of microglia in Toll-like receptor-mediated neuronal injury. Glia.

[101] Ferreira S.A., Romero-Ramos M. (2018). Microglia Response During Parkinson’s Disease: Alpha-Synuclein Intervention. Front. Cell Neurosci..

[102] Harms A.S., Cao S., Rowse A.L., Thome A.D., Li X., Mangieri L.R., Cron R.Q., Shacka J.J., Raman C., Standaert D.G. (2013). MHCII is required for α-synuclein-induced activation of microglia, CD4 T cell proliferation, and dopaminergic neurodegeneration. J. Neurosci..

[103] Gao H.M., Zhang F., Zhou H., Kam W., Wilson B., Hong J.S. (2011). Neuroinflammation and α-synuclein dysfunction potentiate each other, driving chronic progression of neurodegeneration in a mouse model of Parkinson’s disease. Environ. Health Perspect..

[104] Steger M., Tonelli F., Ito G., Davies P., Trost M., Vetter M., Wachter S., Lorentzen E., Duddy G., Wilson S. (2017). Phosphoproteomics reveals that Parkinson’s disease kinase LRRK2 regulates a subset of Rab GTPases. eLife.

[105] West A.B., Moore D.J., Biskup S., Bugayenko A., Smith W.W., Ross C.A., Dawson V.L., Dawson T.M. (2005). Parkinson’s disease-associated mutations in leucine-rich repeat kinase 2 augment kinase activity. Proc. Natl. Acad. Sci. USA.

[106] Di Maio R., Hoffman E.K., Rocha E.M., Keeney M.T., Sanders L.H., De Miranda B.R., Zharikov A., Van Laar A., Stepan A., Lanz T.A. (2018). LRRK2 activation in idiopathic Parkinson’s disease. Sci. Transl. Med..

[107] Hauser D.N., Hastings T.G. (2013). Mitochondrial dysfunction and oxidative stress in Parkinson’s disease and monogenic parkinsonism. Neurobiol. Dis..

[108] Pickrell A.M., Youle R.J. (2015). The roles of PINK1, parkin, and mitochondrial fidelity in Parkinson’s disease. Neuron.

[109] Kopito R.R. (2000). Aggresomes, inclusion bodies and protein aggregation. Trends Cell Biol..

[110] Stefanis L. (2012). α-Synuclein in Parkinson’s disease. Cold Spring Harb. Perspect. Med..

[111] Block M.L., Zecca L., Hong J.S. (2007). Microglia-mediated neurotoxicity: Uncovering the molecular mechanisms. Nat. Rev. Neurosci..

[112] Dias V., Junn E., Mouradian M.M. (2013). The role of oxidative stress in Parkinson’s disease. J. Park. Dis..

[113] Fawcett J.W., Fyhn M., Jendelova P., Kwok J.C.F., Ruzicka J., Sorg B.A. (2022). The extracellular matrix and perineuronal nets in memory. Mol. Psychiatry.

[114] Cenci M.A., Lundblad M. (2006). Post- versus presynaptic plasticity in L-DOPA-induced dyskinesia. J. Neurochem..

[115] Calabresi P., Di Filippo M., Ghiglieri V., Tambasco N., Picconi B. (2010). Levodopa-induced dyskinesias in patients with Parkinson’s disease: Filling the bench-to-bedside gap. Lancet Neurol..

[116] Yong V.W. (2005). Metalloproteinases: Mediators of pathology and regeneration in the CNS. Nat. Rev. Neurosci..

[117] Dominguez-Meijide A., Parrales V., Vasili E., González-Lizárraga F., König A., Lázaro D.F., Lannuzel A., Haik S., Del Bel E., Chehín R. (2021). Doxycycline inhibits α-synuclein-associated pathologies in vitro and in vivo. Neurobiol. Dis..

[118] Mollenhauer B., Zimmermann J., Sixel-Döring F., Focke N.K., Wicke T., Ebentheuer J., Schaumburg M., Lang E., Trautmann E., Zetterberg H. (2016). Monitoring of 30 marker candidates in early Parkinson disease as progression markers. Neurology.

[119] Hall S., Surova Y., Öhrfelt A., Blennow K., Zetterberg H., Hansson O. (2015). Longitudinal measurements of cerebrospinal fluid biomarkers in Parkinson’s disease. Mov. Disord..

[120] Karsdal M.A., Nielsen M.J., Sand J.M., Henriksen K., Genovese F., Bay-Jensen A.C., Smith V., Adamkewicz J.I., Christiansen C., Leeming D.J. (2013). Extracellular matrix remodeling: The common denominator in connective tissue diseases. ASSAY Drug Dev. Technol..

[121] Genovese F., Manresa A.A., Leeming D.J., Karsdal M.A., Boor P. (2014). The extracellular matrix in the kidney: A source of novel non-invasive biomarkers of kidney fibrosis?. Fibrogenesis Tissue Repair.

[122] Chen-Plotkin A.S., Albin R., Alcalay R., Babcock D., Bajaj V., Bowman D., Buko A., Cedarbaum J., Chelsky D., Cookson M.R. (2018). Finding useful biomarkers for Parkinson’s disease. Sci. Transl. Med..

[123] Arya R., Haque A.K.M.A., Shakya H., Billah M.M., Parvin A., Rahman M.M., Sakib K.M., Faruquee H.M., Kumar V., Kim J.J. (2024). Parkinson’s Disease: Biomarkers for Diagnosis and Disease Progression. Int. J. Mol. Sci..

[124] Espay A.J., Schwarzschild M.A., Tanner C.M., Fernandez H.H., Simon D.K., Leverenz J.B., Merola A., Chen-Plotkin A., Brundin P., Kauffman M.A. (2017). Biomarker-driven phenotyping in Parkinson’s disease: A translational missing link in disease-modifying clinical trials. Mov. Disord..

[125] Goldman J.G., Andrews H., Amara A., Naito A., Alcalay R.N., Shaw L.M., Taylor P., Xie T., Tuite P., Henchcliffe C. (2018). Cerebrospinal fluid, plasma, and saliva in the BioFIND study: Relationships among biomarkers and Parkinson’s disease Features. Mov. Disord..

[126] Berg D., Postuma R.B., Adler C.H., Bloem B.R., Chan P., Dubois B., Gasser T., Goetz C.G., Halliday G., Joseph L. (2015). MDS research criteria for prodromal Parkinson’s disease. Mov. Disord..

[127] Avazzadeh S., Baena J.M., Keighron C., Feller-Sanchez Y., Quinlan L.R. (2021). Modelling Parkinson’s Disease: iPSCs towards Better Understanding of Human Pathology. Brain Sci..

